# Modality-specific sensory and decisional carryover effects in duration perception

**DOI:** 10.1186/s12915-023-01547-9

**Published:** 2023-03-08

**Authors:** Baolin Li, Biyao Wang, Adam Zaidel

**Affiliations:** 1 School of Psychology, Shaanxi Normal University, 199 Chang’an South Road, Yanta District, Xi’an, 710062 China; 2 Gonda Multidisciplinary Brain Research Center, Bar-Ilan University, 5290002 Ramat Gan, Israel

**Keywords:** Serial dependence, History effects, Timing, Confirmation bias, Adaptation, Generalization

## Abstract

**Background:**

The brain uses recent history when forming perceptual decisions. This results in carryover effects in perception. Although separate sensory and decisional carryover effects have been shown in many perceptual tasks, their existence and nature in temporal processing are unclear. Here, we investigated whether and how previous stimuli and previous choices affect subsequent duration perception, in vision and audition.

**Results:**

In a series of three experiments, participants were asked to classify visual or auditory stimuli into “shorter” or “longer” duration categories. In experiment 1, visual and auditory stimuli were presented in separate blocks. Results showed that current duration estimates were repelled away from the previous trial’s stimulus duration, but attracted towards the previous choice, in both vision and audition. In experiment 2, visual and auditory stimuli were pseudorandomly presented in one block. We found that sensory and decisional carryover effects occurred only when previous and current stimuli were from the same modality. Experiment 3 further investigated the stimulus dependence of carryover effects within each modality. In this experiment, visual stimuli with different shape topologies (or auditory stimuli with different audio frequencies) were pseudorandomly presented in one visual (or auditory) block. Results demonstrated sensory carryover (within each modality) despite task-irrelevant differences in visual shape topology or audio frequency. By contrast, decisional carryover was reduced (but still present) across different visual topologies and completely absent across different audio frequencies.

**Conclusions:**

These results suggest that serial dependence in duration perception is modality-specific. Moreover, repulsive sensory carryover effects generalize within each modality, whereas attractive decisional carryover effects are contingent on contextual details.

**Supplementary Information:**

The online version contains supplementary material available at 10.1186/s12915-023-01547-9.

## Background

To interact within a dynamic world, our brains need to perceive and interpret temporal information from the environment. However, perceptions of time and duration are prone to distortions and illusions [1, 2]. Among other factors, recent sensory experience exerts a complex influence on duration perception. On the one hand, it can lead to “repulsive” (also known as negative) aftereffects. In this case, repetitive exposure to relatively longer (or shorter) duration stimuli leads to a shortening (or lengthening) of subsequent duration estimates [3, 4]. By contrast, “attractive” (also known as positive) carryover effects have also been found in judgments of duration [5], such as regresssion toward the mean [6]. Also, temporal preparation (readiness to act) is facilitated by timing experience in previous trials [7–10]. Therefore, the specific elements that give rise to different (repulsive and attractive) carryover effects on duration perception require further investigation.

Even single events, such as immediately preceding trials in an experiment, can lead to carryover effects. This phenomenon is often called “serial dependence.” We thus consider serial dependence (resulting from a single or a few immediately preceding trials) a specific subcase of carryover effects, which also include broader timescales. Serial dependence has been observed in discriminating a variety of perceptual features, including visual orientation [11, 12], numerosity [13, 14], motion [15, 16], and more complex features like face identity [17] and attractiveness [18]. Serial dependence is also found in duration perception [19–24]. However, the nature of serial dependence in duration perception remains debated.

The influences of serial dependence are complex and multifactorial. Previous trials typically comprise both stimuli and choices, which can have distinct effects. Correspondingly, the carryover effects of serial dependence can be divided into (at least) two categories: sensory carryover (SC) and decisional carryover (DC). SC refers to the influence of the previous stimulus on the current perception, and DC reflects the influence of the previous choice. Although SC has been previously called perceptual carryover [24], we prefer to use the term SC because perception is a higher-level process of inference, resulting in a “decision” [25], although its underlying mechanisms are still debatable. There is growing evidence that SC and DC lead to opposing effects in serial dependence (repulsive and attractive, respectively). Namely, perceptual decisions are both repulsed from previous stimuli and attracted to previous choices [26–30].

In line with this, Wiener et al. found repulsive SC and attractive DC from previous trials on current duration estimates [24]. Recent studies by Wehrman et al. have further exposed a strong influence of prior experience on duration perception. They found an overall attractive effect of serial dependence, which was attenuated when the response context was changed [22], and implicated DC for this effect [23]. These studies estimated SC (and DC) by sorting responses on the current trials according to the previous trials’ durations (or choices). A limitation of this method is that choices are (by task design) highly correlated with stimulus durations: longer (shorter) stimuli will be judged more often as “longer” (“shorter”). Therefore, sorting trials by previous stimulus duration leads to groups with unbalanced previous choices (and vice versa). Hence, the sorted trials have confounding influences of SC and DC. This can lead to biased estimations of the effects and missed detections (when the two cancel one another out). Therefore, how previous stimuli and previous choices separately affect duration perception is still unclear.

Most research on serial dependence of duration perception to date has focused on the context of a single type of stimulus [19–24]. However, we live in a cluttered environment in which we are commonly confronted with multiple objects and features that need to be perceived and maintained in working memory, simultaneously. Thus, an important open question is how SC and DC of duration perception operate in more complex situations. Specifically, it is unclear whether carryover effects in duration perception are modulated by task-irrelevant stimulus features (such as audio frequency, or the shape of a visual stimulus). Namely, does serial dependence of duration perception still occur when task-irrelevant stimulus features differ across sequential stimuli?

Several studies investigated whether priors for duration perception generalize across different contexts, but the results are inconclusive [31–34]. Roach et al. found that the central tendency in duration reproduction can be explained by a single prior across distinct sensory signals (from different modalities and spatial locations) [33]. However, other studies found that the central tendency of duration estimates was stimulus- and modality-dependent [32, 34]. Thus, the extent to which carryover effects generalize in duration perception requires further research.

Also, regarding other perceptual features (besides duration), whether or not serial dependence generalizes is a matter of debate. Attractive serial dependence occurs in visual orientation perception even across stimuli that differ in color, or spatial location [11, 29, 35, 36]. Similarly, attractive serial dependence regarding face identity transfers across different viewpoints [17]. By contrast, repulsive effects are spatially specific [29, 36]. Together, these studies suggest that for serial dependence, DC, but not SC, might generalize across different stimuli. This notion is in line with a series of studies on serial dependence of stimulus intensity which found that (attractive) DC, but not (repulsive) SC, generalizes across audio and visual modalities [37, 38]. Moreover, DC is not constrained by motor responses [15, 27, 39]. Also, decision categories can be flexibly created [40].

In this study, our first aim was to quantify the separate effects of previous stimuli (i.e., stimulus durations) and previous decisions (i.e., categorical choices regarding duration) in serial dependence of duration perception. To dissociate the separate effects of previous stimuli and previous choices, we applied a probabilistic choice model to our data. This model predicts a participant’s current choices based on several factors, including previous stimuli and previous choices. Since contributions of previous stimuli and previous choices are taken into account concurrently in the model, we could evaluate their separate contributions in an unbiased manner. We predicted that previous stimuli would lead to a repulsive SC, while previous choices would result in an attractive DC for duration perception (in accordance with SC and DC observed regarding other perceptual features). The second aim of the study was to investigate whether the effects would generalize across modalities and task-irrelevant features. We hypothesized that SC would not generalize across modalities and task-irrelevant features (because SC is highly related to the stimulus) and that DC would (because decisions are more high-level, and thus expected to be amodal).

**Fig. 1:**
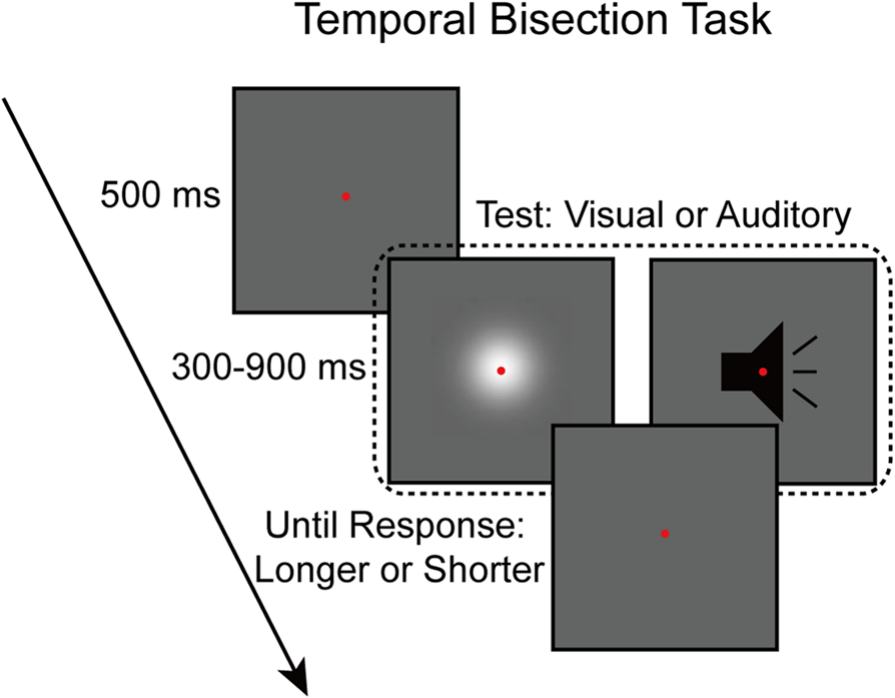
Schematic illustration of a trial. Participants performed visual and auditory temporal bisection tasks, in which they were asked to judge whether the duration of the visual or auditory test stimulus belonged to longer or shorter categories

To test these hypotheses, we conducted three experiments using a temporal bisection task (Fig. 1). In experiment 1, participants were presented with either visual or auditory stimuli from a range of possible durations, and they were asked to classify each stimulus into longer or shorter duration categories. We found repulsive SC and attractive DC in serial dependence of duration estimates for both modalities. We then investigated the cross-modal generalization of these carryover effects in experiment 2, by presenting visual and auditory stimuli interleaved, in one experimental block. Neither SC nor DC transferred between vision and audition. Participants were then tested in experiment 3 with stimuli that differed by a task-irrelevant feature within a single sensory modality (visual or auditory). Within each modality, SC was seen despite differences in task-irrelevant features (visual shape topology or audio frequency). By contrast, DC was reduced for vision, and completely absent for audition, across stimuli that differed in task-irrelevant features. These results suggest that while similar (repulsive SC and attractive DC) components influence duration perception for both modalities, these work independently (and differently) for different modalities. Also, within a specific modality, DC (not SC) is highly sensitive to context.

## Results

### Experiment 1: repulsive sensory carryover and attractive decisional carryover in duration perception

The first experiment aimed to dissociate the unique components of serial dependence: sensory carryover (SC) and decisional carryover (DC), in duration perception. Participants performed a temporal bisection task. This was done separately for vision and audition (in two separate blocks, counterbalanced). We expected to observe two carryover effects in opposite directions: a repulsive SC, indicating that current perceptual decisions are biased away from previous stimuli, and an attractive DC, showing that current perceptual decisions are biased toward previous choices.

### Psychometric results

The main results of this study are attained from the probability choice model (presented in the following section “Model results”). For comparison with previous work (and between methods), we also measured changes in psychometric function PSEs (points of subjective equality) when sorting the data by previous stimuli or previous choices (only for experiment 1). For details regarding this analysis, see the section “Psychometric analysis” in the “Methods” section. However, the limitations of this method should be kept in mind. Specifically, conditioning on the previous stimulus yields an uneven number of longer and shorter previous choices. For example, when the previous trial stimulus was 300 ms, the previous choice (by task design) will more frequently be shorter (vs. longer); conversely, when the previous trial stimulus was 900 ms, the previous choice (by task design) will more frequently be longer (vs. shorter). Similarly, conditioning on previous choices yields an uneven number of previous stimuli. Therefore, this method does not cleanly separate between previous stimuli and previous choices.

The psychometric results are briefly summarized here (for further details, see Additional file 1: Supplementary Results and Figs. S1 and S2) [41–43]. In summary, we found no significant changes in PSE when sorting the data by previous stimuli ($\frac{\delta \textrm{PSE}}{\delta \left(\textrm{prev}\_\textrm{stim}\right)}$, see the section “Psychometric analysis” in the “Methods” section), neither for visual (*t* (23) = 2.0, *p* = 0.25, Cohen’s *d* = 0.40) nor for auditory (*t* (23) = 1.0, *p* = 1.00, Cohen’s *d* = 0.20) stimuli (Additional file 1: Fig. S1, E). However, the changes in PSE by prior choices, ΔPSE_prev_choice_ (see the section “Psychometric analysis” in the “Methods” section), were significantly larger than zero for both visual (*t* (23) = 5.0, *p* < 0.001, Cohen’s *d* = 1.01) and auditory (*t* (23) = 4.0, *p* = 0.003, Cohen’s *d* = 0.81) stimuli (Additional file 1: Fig. S1, F). These results ostensibly suggest an attractive DC and no observable SC in serial dependence of duration perception. However, because the previous stimulus and previous choice effects are not well separated in this analysis (as described above), and the underlying SC and DC may be in opposite directions, these results could misattribute and/or underestimate the effects.

### Model results

**Fig. 2:**
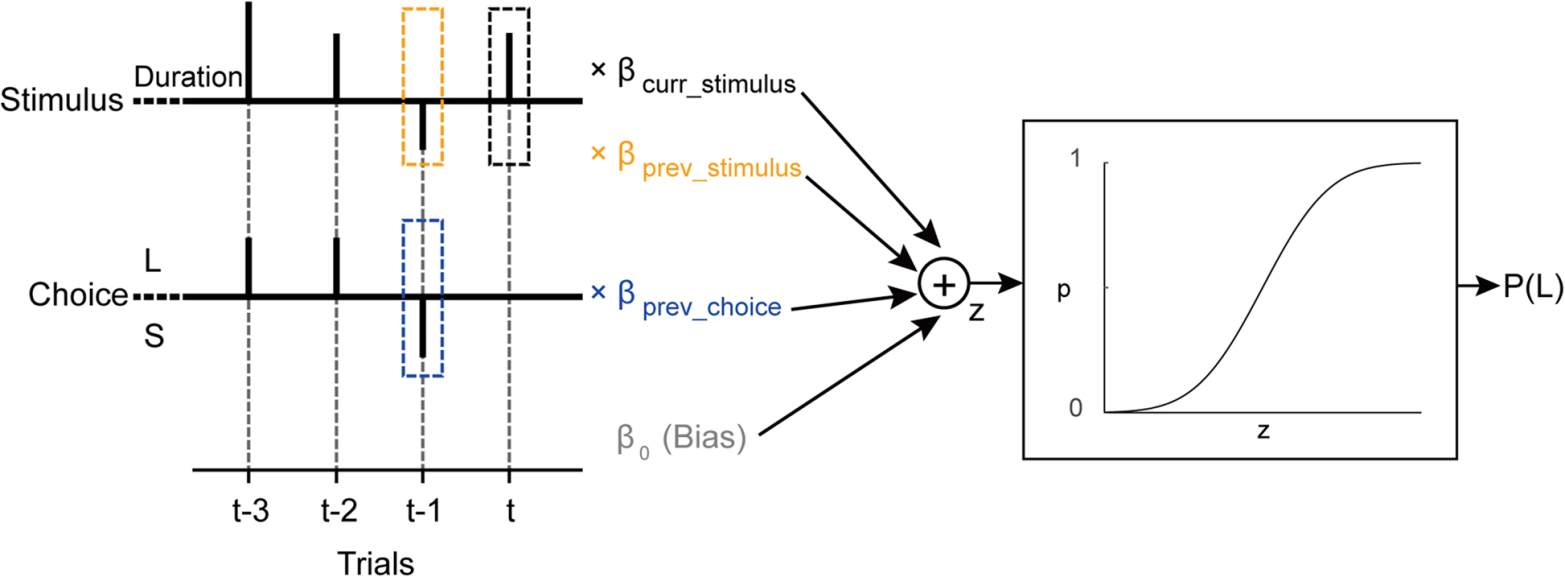
Schematic illustration of the probabilistic choice model. The participant’s perceptual choice is modeled with four predictors: (1) the current stimulus duration (*β*_curr _ stimulus_), (2) the previous stimulus duration (*β*_prev _ stimulus_), (3) the previous choice (*β*_prev _ choice_), and (4) the baseline bias (*β*_0_). Black stems on the stimulus and choice axes depict the stimulus durations and choices, respectively. The current stimulus duration (on trial *t*) is marked by a black dashed box. The stimulus duration and choice from the previous trial (*t*-1) are marked by orange and blue dashed boxes, respectively. The linear combination (z) is passed through a logistic function to yield the probability for choosing longer, P(L) on trial *t*

**Fig. 3:**
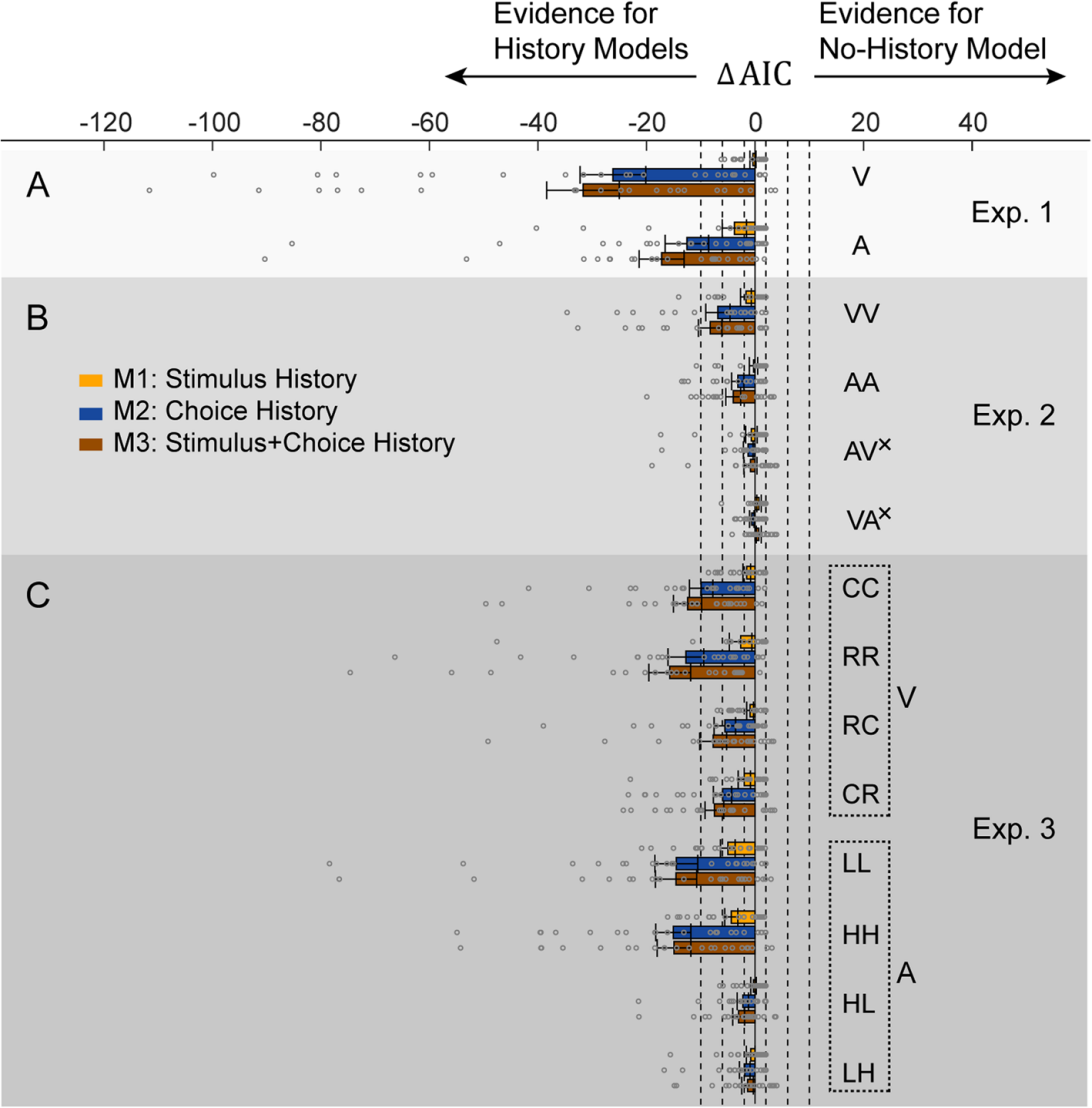
AIC Model comparisons. ΔAIC values for each of the history models (*M*_1 − 3_, color coded) vs. the no-history model (*M*_0_), applied to the data from (A) experiment 1, (B) experiment 2, and (C) experiment 3, per condition (marked by one- or two-letter codes, explained in Table 1; ^×^ marks cross-modal conditions). The bars represent group means and gray circles represent the individual participants’ ΔAIC values, for each experiment and condition. Error bars indicate standard errors of the mean. ΔAIC magnitudes between 2 and 6, between 6 and 10, and > 10 are considered positive, strong, and very strong evidence, respectively, for the model with the lower AIC value. These boundaries (ΔAIC = ±2, ±6, and ±10) are marked by vertical dashed lines. Similar model comparisons using BIC are presented in Additional file 1: Fig. S3

We applied the probability choice model (with and without history effects; Fig. 2) and performed model comparisons to evaluate whether the influences of previous information (stimuli and choices from previous trials) on current perceptual decisions were substantial (see the section “Probabilistic choice model” in the “Methods” section). To compare models, we calculated the AIC (Akaike information criterion, Eq. 3) for each model and then calculated the difference in AIC (ΔAIC, Eq. 4) between each of the history models (*M*_1 − 3_) and the “no-history” model (*M*_0_), separately for the visual and auditory tasks. Overall, ΔAICs were negative both for the visual and auditory tasks (Fig. 3A), suggesting better model fits when taking history into account. More specifically, the “stimulus and choice history” model performed best (ΔAIC_M3_= −31.7 for visual and −17.2 for auditory) followed by the “choice history” model (ΔAIC_M2_= −26.2 and −12.6, respectively), while the “stimulus history” model was marginally better than *M*_0_ (ΔAIC_M1_= −0.4 and −3.8, respectively). Similarly, BIC (Bayesian information criterion, Eq. 5) model comparisons also suggest better model fits when taking previous choices (*M*_2_) or both previous stimuli and previous choices (*M*_3_) into account (Additional file 1: Fig. S3, A). Moreover, *M*_3_ better explained participant’s choices (vs. *M*_0_) for the vast majority of participants (88% for visual and 83% for auditory, likelihood ratio test; Additional file 1: Fig. S4, C). These results provide strong support for taking prior history into account. Because *M*_3_ simultaneously takes into account both previous stimuli and previous choices, we focused on the parameters of *M*_3_ for further analysis. This approach was also justified by the model comparison results—the largest ΔAIC magnitudes were seen for *M*_3_, with differences vs. *M*_2_ and *M*_1_ > 4 (for both modalities).

**Fig. 4:**
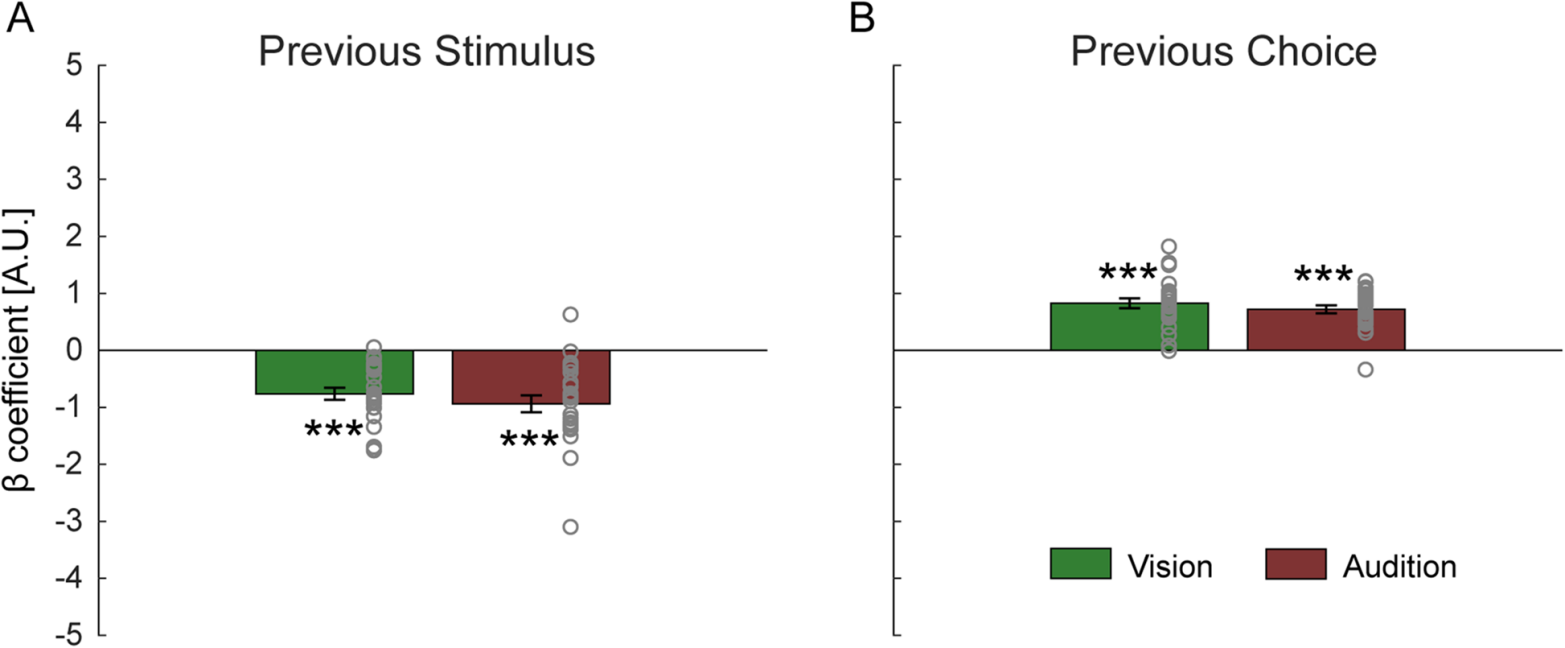
History-related model parameters from experiment 1. Beta coefficients for predictors: **A** previous stimulus (*β*_prev _ stimulus_) and **B** previous choice (*β*_prev _ choice_), from the *M*_3_ (stimulus and choice history) model fits, in vision (green bars) and audition (maroon bars). Bars represent group means, error bars indicate standard errors of the mean, and gray circles represent individual participants’ values. ***p < 0.001, Bonferroni corrected by multiplying the raw *p*-values by four

To investigate the separate effects of previous stimuli and previous choices, two model parameters (*β*_prev _ stimulus_, *β*_prev _ choice_ taken from the *M*_3_ fits, see Eq. 2) were statistically analyzed (Fig. 4). For both the visual and auditory tasks, *β*_prev _ stimulus_ coefficients were significantly negative (vision: *t* (23) = −7.2, *p* < 0.001, Cohen’s *d* = −1.47; audition: *t* (23) = −6.3, *p* < 0.001 Cohen’s *d* = −1.30), while *β*_prev _ choice_ coefficients were significantly positive (vision: *t* (23) = 9.4, *p* < 0.001, Cohen’s *d* = 1.92; audition: *t* (23) = 10.2, *p* < 0.001, Cohen’s *d* = 2.08). Paired-samples *t*-tests showed that neither the *β*_prev _ stimulus_ coefficient (*t* (23) = 1.1, *p* = 1.00, Cohen’s *d* = 0.23) nor the *β*_prev _ choice_ coefficient (*t* (23) = 1.1, *p* = 1.00, Cohen’s *d* = 0.23) was significantly different between vision and audition. These results indicate that both a repulsive effect of previous stimuli and an attractive effect of previous choices are present in duration perception. And that these effects are comparable in vision and audition.

Experiment 1 investigated whether and how previous stimuli and previous choices affect current duration estimates. We applied two different methods of analysis: (i) using psychometric functions, conditioned either on previous stimuli or on previous choices, and (ii) fitting a logistic regression model that takes into account both the effects of previous stimuli and previous choices, at the same time. The psychometric results suggested attractive DC in duration perception, similar to the results of previous studies [23, 24]. However, this method does not fully dissociate the separate effects of previous choices from those of previous stimuli. Because stimulus durations and choices are correlated (by task design), the effects of previous stimuli and previous choices are confounded in the psychometrics. Moreover, previous stimuli and previous choices often have opposite influences on subsequent decisions [12, 26, 29, 30]. Thus, the effects of previous stimuli and previous choices could counteract one another. Hence, not finding SC in the psychometric analysis is inconclusive. Indeed, the model results exposed significant contributions of both previous stimuli and previous choices on the current perceptual decision, both in vision and audition. These results indicate that duration perception is subject to both (repulsive) SC and (attractive) DC as seen for many other sensory dimensions, besides duration.

### Experiment 2: both sensory and decisional carryovers are modality-specific

Experiment 1 verified the presence of both SC and DC in duration perception, for both vision and audition. However, it is unclear whether these effects are limited to a single modality or generalize across modalities. To test this, in experiment 2, participants were presented visual and auditory stimuli interleaved pseudorandomly in one block. Given the limitations of $\frac{\delta \textrm{PSE}}{\delta \left(\textrm{prev}\_\textrm{stim}\right)}$ and ΔPSE_prev_choice_ measures (as described in experiment 1 “Psychometric results” section in the “Results” section), we did not calculate these measures for experiments 2 and 3 (average psychometric curves for these experiments are presented in Additional file 1: Fig. S5). Rather, we focus on the logistic regression model fits.

**Fig. 5:**
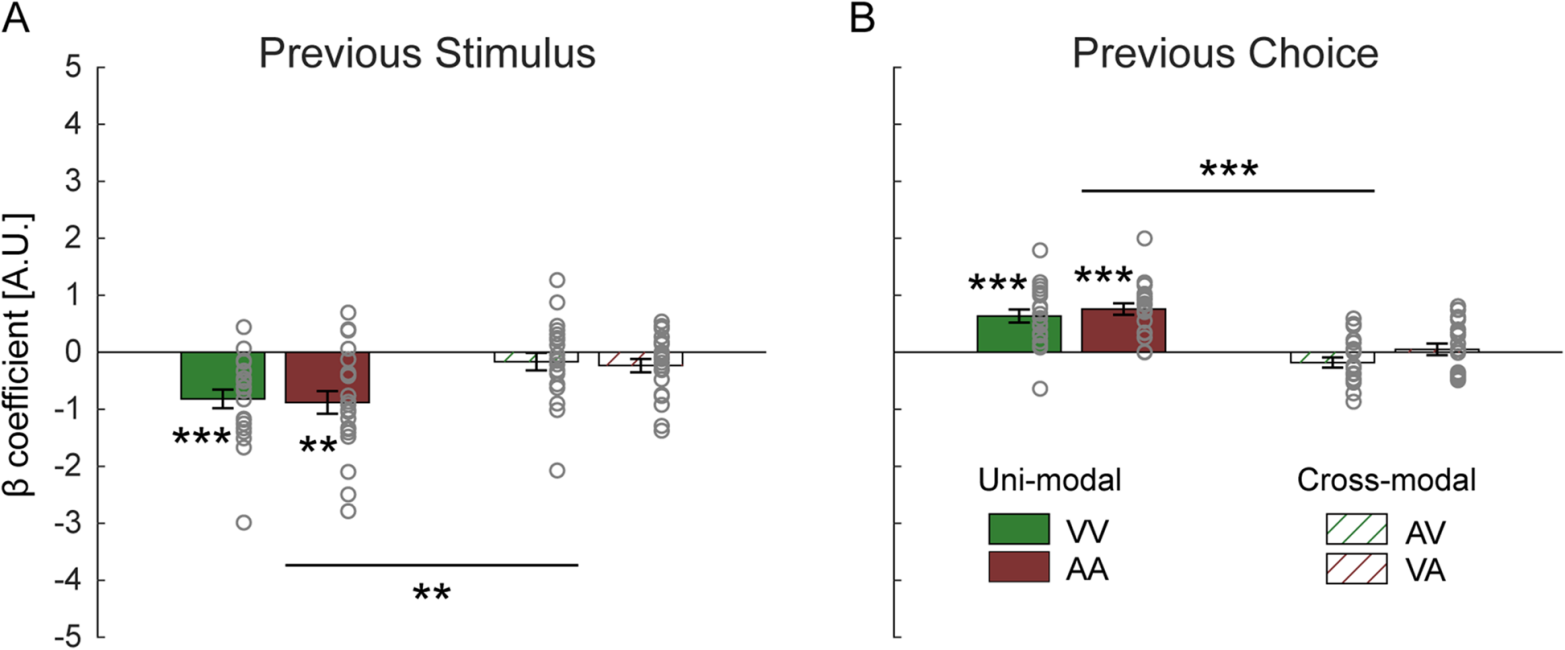
History-related model parameters from experiment 2. Beta coefficients for predictors: **A** previous stimulus (*β*_prev _ stimulus_) and **B** previous choice (*β*_prev _ choice_), from the *M*_3_ (stimulus and choice history) model fits in different conditions (V visual, A auditory; see full condition details in Table 1 part B). Bars represent group means, error bars indicate standard errors of the mean, and gray circles represent individual participants’ values. ***p < 0.001, **p < 0.01, Bonferroni corrected by multiplying the raw *p*-values by four

The trials were divided into four conditions, according to the modalities of the current and previous trials’ stimuli (see Table 1 part B). The data for each condition was fitted, per participant, with the probabilistic choice model similar to experiment 1. In experiment 2, we found significant influences of previous stimuli and previous choices in the uni-modal (VV and AA), but not in the cross-modal (AV and VA) contexts (Fig. 5). Specifically, the *β*_prev _ stimulus_ coefficients were significantly negative in conditions VV (*t* (20) = −5.0, *p* < 0.001, Cohen’s *d* = −1.10) and AA (*t* (20) = −4.4, *p* = 0.001, Cohen’s *d* = −0.96), but did not differ significantly from zero in conditions AV (*t* (20) = −1.1, *p* = 1.00, Cohen’s *d* = −0.23) and VA (*t* (20) = −2.0, *p* = 0.24, Cohen’s *d* = −0.44). The 2 (modality: vision, audition) × 2 (context: uni-modal, cross-modal) repeated-measures ANOVA (analysis of variance) showed a significant main effect of context (*F* (1, 20) = 13.2, *p* = 0.007, *η*_p_^2^ = 0.40), with a greater influence of previous stimuli in the uni-modal vs. cross-modal context. Neither a main effect of modality nor an interaction between modality and context was significant (both *p*-values > 0.05).

**Table 1:** Experimental conditions

	Experiment #	Abbreviation	Previous trial Modality/stimulus		Current trial Modality/stimulus		Context
**A**	1	V	Visual/Gaussian blob				Uni-modal
		A	Auditory/White noise				
**B**	2	VV	Visual/Gaussian blob				Uni-modal
		AA	Auditory/white noise				
		AV	Auditory/white noise		Visual/Gaussian blob		Cross-modal
		VA	Visual/Gaussian blob		Auditory/white noise		
**C**	3	CC	Visual	Circle	Visual	Circle	Consistent-stimulus
		RR		Ring		Ring	
		RC		Ring		Circle	Inconsistent-stimulus
		CR		Circle		Ring	
		LL	Auditory	Low	Auditory	Low	Consistent-stimulus
		HH		High		High	
		HL		High		Low	Inconsistent-stimulus
		LH		Low		High

The *β*_prev _ choice_ coefficients were significantly positive in conditions VV (*t* (20) = 5.6, *p* < 0.001, Cohen’s *d* = 1.21) and AA (*t* (20) = 7.5, *p* < 0.001, Cohen’s *d* = 1.64), but did not differ significantly from zero in conditions AV (*t* (20) = −2.0, *p* = 0.22, Cohen’s *d* = −0.45) and VA (*t* (20) = 0.5, *p* = 1.00, Cohen’s *d* = 0.11). The repeated-measures ANOVA showed significantly larger *β*_prev _ choice_ coefficients in the uni-modal vs. cross-modal contexts (*F* (1, 20) = 41.8, *p* < 0.001, *η*_p_^2^ = 0.68). Neither a main effect of modality nor an interaction between modality and context was significant (both *p*-values > 0.05). These results indicate that both SC and DC are modality-specific.

The AIC model comparisons provide further evidence for modality-specific carryover effects in duration perception (Fig. 3B). In the uni-modal conditions, the stimulus and choice history (*M*_3_) and choice history (*M*_2_) models performed better than the no-history model (*M*_0_): ΔAIC_M3_= −8.3 and ΔAIC_M2_= −6.9 for VV and ΔAIC_M3_= −4.0 and ΔAIC_M2_= −3.2 for AA. The stimulus history model (*M*_1_) showed marginal differences (ΔAIC_M1_= −1.7 and −0.3 for VV and AA, respectively). In the cross-model conditions, all ΔAIC magnitudes were marginal (ΔAIC_M3_= −0.9, ΔAIC_M2_= −1.3, ΔAIC_M1_= −0.7 for AV, and ΔAIC_M3_= 0.7, ΔAIC_M2_= −0.6, ΔAIC_M1_= 0.7 for VA), indicating no substantial advantage for the models with history. BIC model comparisons show a preference for *M*_0_ over *M*_3_ in the cross-modal conditions (Additional file 1: Fig. S3, B). Similarly, *M*_0_ better explained the participant’s choices (vs. *M*_3_) for the majority of participants (81% for AV and 95% for VA, likelihood ratio test; Additional file 1: Fig. S4, C).

Experiment 2 tested cross-modal generalization of the carryover effects in serial dependence. The results indicate that both SC and DC are contingent on modality. That is, serial dependence in duration perception occurred when previous and current stimuli were from the same modality, but not when they were from different modalities. These results are consistent with previous studies suggesting modality-specific timing mechanisms [44].

### Experiment 3: decisional, but not sensory, carryover is contingent on stimulus details

Experiment 2 revealed that both SC and DC effects in duration perception are modality-specific. This suggests modality-specific mechanisms of serial dependence. However, it is unclear whether these aspects of serial dependence generalize within a sensory modality. Are SC and DC in duration perception stimulus-specific, within a sensory modality? To investigate this question, we conducted experiment 3, which presented visual stimuli with different topologies or auditory stimuli with different sound frequencies. The different stimuli within a modality were presented pseudorandomly in dedicated visual and auditory blocks. For each of the four conditions, per modality (see Table 1 part C), model coefficients were extracted, similar to experiment 2.

### Carryover effects in the visual task

**Fig. 6:**
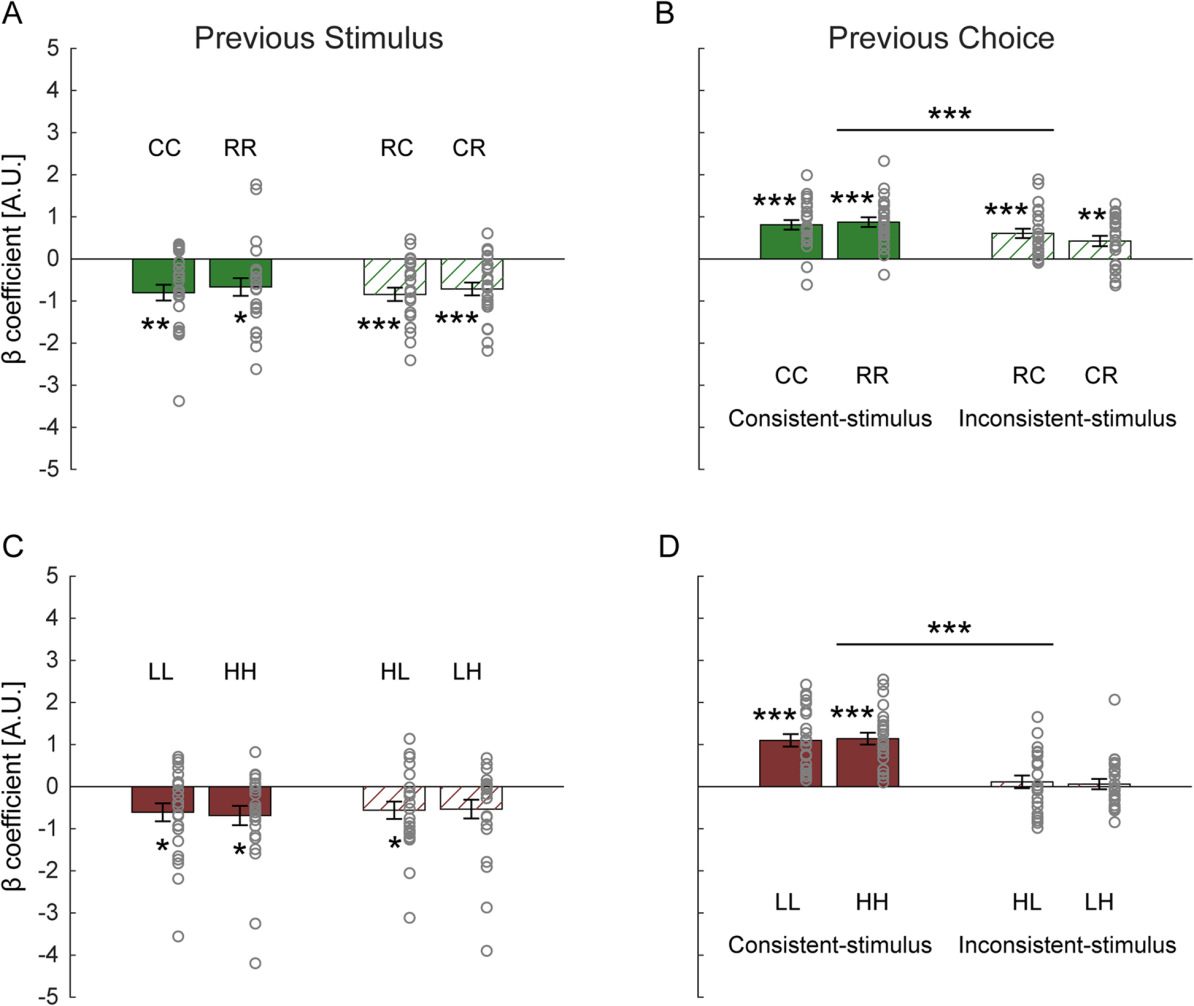
History-related model parameters from experiment 3. Beta coefficients for predictors: **A**, **C** previous stimulus (*β*_prev _ stimulus_) and **B**, **D** previous choice (*β*_prev _ choice_), from the *M*_3_ (stimulus and choice history) model fits in different conditions (C circle, R ring, L low-pitch, H high-pitch; see full condition details in Table 1 part C). Bars represent group means, error bars indicate standard errors of the mean, and gray circles represent individual participants’ values. ***p < 0.001, **p < 0.01, *p < 0.05, Bonferroni corrected by multiplying the raw *p*-values by four

One-sample *t*-tests showed that the *β*_prev _ stimulus_ coefficients were significantly negative in all four visual conditions (CC: *t* (23) = −4.3, *p* = 0.001, Cohen’s *d* = −0.87; RR: *t* (23) = -3.2, *p* = 0.017, Cohen’s *d* = −0.65; RC: *t* (23) = −5.3, *p* < 0.001, Cohen’s *d* = −1.09; CR: *t* (23) = −4.7, *p* < 0.001, Cohen’s *d* = −0.96; Fig. 6A). Moreover, there were no significant main effects, nor an interaction in the 2 (stimulus: circle, ring) × 2 (context: consistent-stimulus, inconsistent-stimulus) repeated-measures ANOVA (all *p*-values > 0.05). These results suggest that for vision, previous stimuli modulate the current perceptual decision (in a repulsive manner) even across visual objects with different topologies.

Next, we tested the effect of previous choices quantified by the *β*_prev _ choice_ coefficient. We observed that all the *β*_prev _ choice_ coefficients were significantly positive in all four visual conditions (CC: *t* (23) = 7.1, *p* < 0.001, Cohen’s *d* = 1.46; RR: *t* (23) = 7.6, *p* < 0.001, Cohen’s *d* = 1.55; RC: *t* (23) = 5.4, *p* < 0.001, Cohen’s *d* = 1.11; CR: *t* (23) = 3.5, *p* = 0.008, Cohen’s *d* = 0.72; Fig. 6B). These results suggest that (at least part of) the effect of previous choices generalizes across different visual stimuli. The repeated-measures ANOVA revealed a significant main effect of context (*F* (1, 23) = 23.2, *p* < 0.001, *η*_p_^2^ = 0.50), showing a larger effect of previous choices in the consistent-stimulus (vs. inconsistent-stimulus) context. Neither the main effect of stimulus nor the interaction between stimulus and context was significant (both *p*-values > 0.05). This further suggests that DC is modulated by context. In other words, DC in vision is reduced when object topology is changed.

The model comparisons showed negative ΔAICs (in favor of the models with history) in all four visual conditions (Fig. 3C). Once again, the stimulus and choice history model (*M*_3_) performed best and was substantially better than *M*_0_ in both the consistent-stimulus context (ΔAIC_M3_= −12.4 for CC and −15.7 for RR) and the inconsistent-stimulus context (ΔAIC_M3_= −7.7 for RC and −7.5 for CR). The choice history model (*M*_2_) was also better than *M*_0_ (ΔAIC_M2_= −10.0, −12.8, −5.6, and −6.0, for CC, RR, RC, and CR, respectively) and the stimulus history model (*M*_1_) was marginally better than *M*_0_ (ΔAIC_M1_= −1.5, −2.7, −0.9 and −2.0, for CC, RR, RC, and CR, respectively). Although BIC model comparisons show an advantage of history (*M*_3_ and *M*_2_, over *M*_0_) only for the consistent-stimulus, but not the inconsistent-stimulus, conditions (Additional file 1: Fig. S3, C), *M*_3_ better explained most participant’s choices (vs. *M*_0_) in all four conditions (88% for CC, 96% for RR, 63% for RC, and 67% for CR, likelihood ratio test; Additional file 1: Fig. S4, C). These results provide evidence that trial history should be taken into account, even when visual objects differ in topology.

### Carryover effects in the auditory task

Regarding SC, *β*_prev _ stimulus_ coefficients were significantly negative in conditions LL (*t* (23) = −2.9, *p* = 0.035, Cohen’s *d* = −0.58) and HH (*t* (23) = −3.0, *p* = 0.026, Cohen’s *d* = −0.61; Fig. 6C). It was also significantly negative in condition HL (*t* (23) = −2.7, *p* = 0.049, Cohen’s *d* = −0.56) and tended to be negative (but this did not reach significance) in condition LH (*t* (23) = −2.4, *p* = 0.10, Cohen’s *d* = −0.49). Because the LH result was borderline (after correction for multiple comparisons), we also calculated the Bayes factor (BF_10_) of the alternative hypothesis (H_1_: a significant effect of previous stimuli) against the null hypothesis (H_0_: no significant effect of previous stimuli), using a Bayesian one-sample *t*-test. BF_10_ value in condition LH was 2.2, providing some evidence in favor of H_1_ [45]. The 2 (stimulus: low-pitch, high-pitch) × 2 (context: consistent-stimuli, inconsistent-stimuli) repeated-measures ANOVA showed no significant main effects, nor an interaction (all *p*-values > 0.05). These results together suggest that SC in audition is not contingent on audio frequency.

Regarding DC, the *β*_prev _ choice_ coefficients were significantly positive only in the consistent-stimuli conditions: LL (*t* (23) = 7.4, *p* < 0.001, Cohen’s *d* = 1.51) and HH (*t* (23) = 8.1, *p* < 0.001, Cohen’s *d* = 1.65). By contrast, they did not differ significantly from zero in the inconsistent-stimuli conditions: HL (*t* (23) = 0.8, *p* = 1.00, Cohen’s *d* = 0.15) and LH (*t* (23) = 0.5, *p* = 1.00, Cohen’s *d* = 0.10; Fig. 6D). This was further supported by the repeated-measures ANOVA, which showed a significant main effect of context: *β*_prev _ choice_ coefficients were significantly larger in the consistent-stimulus vs. inconsistent-stimulus contexts (*F* (1, 23) = 59.9, *p* < 0.001, *η*_p_^2^ = 0.72). There was no significant main effect of stimulus nor an interaction (both *p*-values > 0.05). These results indicate that the effect of previous choices does not transfer between low- and high-pitch stimuli. DC effects are thus frequency-specific.

For the auditory task, AIC model comparisons (Fig. 3C) indicate a substantial improvement when taking history into account primarily for the consistent-stimulus context (ΔAIC_M3_= −14.6 and −15.0, ΔAIC_M2_= −14.5 and −15.1, ΔAIC_M1_= −5.0 and −4.4, for LL and HH, respectively). For the inconsistent-stimulus context, only a marginal advantage was seen when taking history into account (ΔAIC_M3_= −3.0 and −1.4, ΔAIC_M2_= −2.3 and −1.9, ΔAIC_M1_ = −0.3 and −0.8 for HL and LH, respectively). Similarly, BIC model comparisons suggest a clear advantage of taking history into account for the consistent-stimulus conditions (*M*_3_ and *M*_2_, vs. *M*_0_), with a slight advantage of *M*_0_ over the history models for the inconsistent-stimulus conditions (Additional file 1: Fig. S3, C). Also, *M*_3_ provided better fits (vs. *M*_0_) for the most participants in the consistent-stimulus conditions (75% for LL and 79% for HH), but not in the inconsistent-stimulus conditions (42% for HL and 33% for LH, likelihood ratio test; Additional file 1: Fig. S4, C). These results suggest that it is less important to take trial history into account across auditory stimuli with different frequencies.

Experiment 3 tested stimulus specificity (within each sensory modality) of serial dependence in duration perception. Model comparisons exposed the importance of taking trial history into account for visual stimuli even when they differed across trials, but indicated a limited influence of trial history across different auditory stimuli. Thus, carryover effects of duration perception generalize across different visual stimuli, but this happens to a lesser degree across different auditory stimuli. This difference seems primarily related to changes in DC. For both modalities, while SC was largely unchanged by stimulus context, DC was significantly affected. But, this was much more pronounced for auditory stimuli. Namely, DC was strong for consistent auditory stimuli, but largely absent across auditory stimuli that differed in pitch. By contrast, DC was only reduced (but nonetheless still significantly present) across different visual stimuli.

## Discussion

This study investigated the effects of previous stimuli and previous choices on subsequent temporal decisions, in vision and audition. For this, we designed and ran three experiments to test the existence, magnitude, direction, and generalization of these aspects of serial dependence in duration perception. We found that both previous stimuli and previous choices modulated current perceptual decisions about stimulus duration, but in opposite directions. Specifically, we found repulsive SC and attractive DC, both in vision and audition. However, both SC and DC were modality-specific (they did not transfer cross-modally). Moreover, we found different levels of stimulus specificity within vision and audition. While SC was invariant to changes in stimulus details (visual object topography or audio frequency) for both modalities, DC was contingent on stimulus details. Across different visual stimuli, DC was reduced (but nonetheless present). Across different audio stimuli, DC was reduced to the extent that it could no longer be detected. These results suggest that serial dependence of duration perception (both SC and DC) is modality-specific and that even within a specific modality, context makes a difference (particularly for DC).

Dyjas et al. proposed that when discriminating stimulus duration, people dynamically update their internal reference on a trial-by-trial basis (the Internal Reference Model, IRM) [20]. They tested this in a two-interval discrimination task—i.e., on each trial, both a standard (fixed reference) and a comparison (variable test) stimulus duration were presented. According to the IRM, a weighted combination of the previous trial’s internal reference and the first stimulus on the current trial provides a new internal reference, against which the second interval (on the current trial) is compared. The IRM accounts for many phenomena, including order effects (differences in discrimination sensitivity when the standard/comparison is presented first/second) and attractive carryover effects in the two-interval paradigm [19, 20]. The IRM can also explain the repulsive SC we found here—for single-interval discriminations, the internal reference is attracted to the previous trial’s stimulus duration, leading to repulsion of the current estimate (away from the previous stimulus). This idea is also consistent with Bayesian models that suggest iterative updating of a memory prior [24, 46]. However, the IRM does not take into account possible effects that the second interval can have on subsequent trials (in two-interval trials), and it does not take into account influences of prior decisions. Thus, further theoretical work is needed to combine the models into a comprehensive account.

Consistent with previous studies [37, 38], we found that SC in duration perception was modality-specific—namely SC was not seen between successive visual and auditory (or auditory and visual) stimuli. This extends upon previous studies which suggest independent sensory priors for different modalities in temporal processing [32, 34]. However, within a specific modality (visual or auditory), SC occurred even across stimuli with different attributes (visual topology or auditory frequency). These results suggest that duration estimates are updated per modality and that this process generalizes across stimuli (within a modality) that differ in irrelevant features.

Repulsive SC in serial dependence is reminiscent of the repulsive duration aftereffect seen after many repetitions of a stimulus [3, 4]. Thus, they might share common mechanisms. A previous study found that the duration aftereffect following many repetitions of a stimulus is modality-specific and contingent on audio frequency but not contingent on visual orientation [4]. This shares many similarities with our results (modality-specific carryover effects and carryover between different visual stimuli). However, there are also differences: the present study (which separated SC from DC) found SC across auditory frequencies. Thus, for the auditory perception of duration, SC from serial dependence and adaptation to many repetitions of a stimulus may operate at different levels of sensory processing.

It has been proposed that sensory adaptation operates at varied time scales in different cortical areas [47, 48]. For example, long-term adaptation (i.e., to stimuli presented for a long time) to an oriented visual pattern produced fMRI adaptation across multiple visual areas, including both lower and higher visual cortices, while short-term adaptation showed neural adaptation only in higher visual cortices [48]. Thus, the specificity of aftereffects could be dependent on the adaptation duration. Indeed, previous studies have shown that the position-specific face aftereffect was observed only after long-term (e.g., 5000 ms) adaptation, while short-term (e.g., 500 ms) adaptation resulted in a position-invariant face aftereffect [49, 50]. Accordingly, it is possible that the stimulus invariant SC that we observed in serial dependence occurs primarily in higher-level brain areas.

According to the channel-based model of duration perception, the human brain is endowed with duration detectors with overlapping tuning properties [3]. Accordingly, adaptation to duration diminishes the responses of the relevant detectors, resulting in a repulsive duration aftereffect. Consistent with this model, neuroimaging studies in humans have shown the existence of neuronal representations in the right supramarginal gyrus (SMG) that selectively adapt to different durations [51, 52]. Moreover, topographically organized maps of duration tuning (chronotopic maps) were recently found in the human supplementary motor area (SMA), which are modulated by temporal context and linked to behavioral performance in temporal tasks [53, 54]. These studies suggest that duration channels, located in later processing stages, are linked to duration perception. These duration channels offer a potential neural basis for the observed repulsive SC.

The attractive DC observed here in duration discriminations is consistent with prevalent findings of decision inertia in perceptual choices [12, 15, 26, 55–57]. Previous studies have suggested that attractive DC can be accounted for by “an autonomous learning mechanism” in which the choice made on the previous trial updates the choice likelihood for the current trial [15]. In this framework, the actual choice serves in lieu of feedback. This model, together with our findings that DC is modality-specific and context-dependent, suggests that the brain maintains separate choice likelihoods for (and even within) different modalities.

Previous studies have suggested that associative areas in the posterior parietal cortex (PPC) and prefrontal cortex (PFC) carry history information that seems to play a causal role in choice history biases [58, 59]. Specifically, attractive DC might be underscored by brain areas involved in perceptual decision-making, such as posterior cingulate cortex (CGp), PPC, frontal eye field (FEF), and dorsolateral prefrontal cortex (dlPFC) [15, 25, 55, 60]. Moreover, it is possible that interactions between these (DC related) areas and those implicated in SC (SMG and SMA, described above) underlie the process of stimulus duration perception.

The attractive DC exhibited clear context modulation. Both the context of sensory modality and the stimulus context within a modality modulated DC. Feigin et al. found that DC in location perception was modulated by the similarity of sensory attributes and action-related attributes [27]. This suggests that relevance across perceptual decisions is a key factor for DC. In line with these results regarding location perception, also here regarding duration perception, we found that DC still occurred (but to a lesser degree) across visual stimuli with differences in irrelevant stimulus attributes. Indeed, classification of stimuli depends on their degree of similarity, and carryover effects are modulated by stimulus category [40]. In fact, in experiments 2 and 3, participants were directly instructed to compare the test stimulus with its corresponding reference (visual or auditory in experiment 2; circle or ring in the visual task of experiment 3; low-pitch or high-pitch in the auditory task of experiment 3). This could have encouraged participants to form separate modality and stimulus categories, which might have increased the modality/stimulus specificity of DC.

Moreover, we observed modality differences in generalizations for DC. That is, DC partially generalized across visual stimuli with different shapes, but it did not generalize across audio stimuli with different frequencies at all. This could be related to differences of temporal processing between vision and audition. Previous studies have suggested that audition is the dominant modality for temporal processing [41–43, 61]. Thus, changing visual shape may not be equal to changing audio frequency for temporal processing. Specifically, as a basic element of sound, audio frequency itself is a temporal property and it is salient information in temporal processing, which could facilitate the category formation. By contrast, although individuals are sensitive to topological changes in vision [62, 63], topology is not directly involved in temporal processing, and visual temporal information is considered to be a higher-level stimulus attribute [4, 52, 64]. Thus, participants may tend to classify different visual stimuli into the same category for temporal decisions. This may promote the generalization of DC between stimuli with different shape topologies.

Roach et al. found that priors can generalize across modalities specifically when they are acted upon in the same way [33]. In that study, when the participants reported their duration estimates, for both visual and auditory stimuli, by manually reproducing them (holding down a button on the keyboard for the duration), cross-modal priors were seen. However, when the stimuli were coupled with different motor outputs, multiple priors were formed. Our findings (of no cross-modal effects) suggest that a binary two-alternative choice button press is not enough to create a generalized cross-modal prior. The generalization effects seen in Roach et al. thus likely result from the act of reproducing durations (which was the same for both modalities) [33]. Accordingly, subsequent studies with the temporal discrimination task showed that temporal central tendency effects did not transfer between vision and audition [32, 34].

In contrast to these findings, Ellinghaus et al. found evidence for an amodal reference of time in the two-interval duration comparison task [31]. Specifically, they found a comparable influence of order (whether the standard or comparison stimulus came first) on discrimination sensitivity, even when the modalities were alternating. This conclusion relies on the IRM (which predicts a reduced influence for modality-specific priors). However, other Bayesian models that apply priors to both intervals (in which the first is influenced more than the second, because it is further back in time [65]) can predict similar results without the need to assume an amodal prior. Nevertheless, Ellinghaus et al. in conjunction with Roach et al. indicate that amodal priors of duration perception can be formed in specific contexts [31, 33]. Thus, while the brain has the capacity to form separate duration priors per modality, amodal duration priors can likely also be applied.

The relationship between our results and serial effects in temporal preparation is not trivial. While both probe timing processing in the brain, the tasks, measurements, and stimulus contingencies differ (e.g., reaction times are often the measure of interest in temporal preparation vs. perceptual decisions about a stimulus duration per se, in our task). A possible connection relates to the “foreperiod” in temporal preparation (the pause between the ready signal and the go stimulus) which manifests facilitatory carryover effects [7–10]. However, duration estimates of a foreperiod (a pause, preceding the target stimulus) and duration estimates of the sensory stimulus itself (as in our case) might elicit different phenomena of serial dependence. The notion that repulsive SC is stimulus/sensory driven might predict that it may be seen specifically in duration estimates of sensory stimuli, and maybe not observed in foreperiods (temporal preparation). Finally, other differences, such as the implicit or explicit nature of timing [7], might affect carryover effects in temporal perception.

## Conclusions

In summary, we have shown here that both previous stimuli and previous choices affect subsequent temporal decisions about duration, resulting in repulsive SC and attractive DC. Moreover, both SC and DC in duration perception are modality-specific, namely they do not generalize across visual and auditory cues. Generalization within a specific sensory modality differed for SC and DC. While SC was not contingent on visual shape topology or on audio frequency (it generalized across these features, within a given modality), DC was modulated by these task-irrelevant features. DC was reduced across visual stimuli with different object topologies and completely absent across auditory stimuli with different frequencies. The present results expose different modes of operation of SC and DC in duration perception. They further underline modality differences between visual and auditory processing of duration. The findings contribute to a growing body of research aimed at understanding the formation of sensory and decisional priors in different contexts in duration perception.

## Methods

### Participants

Three groups of 24 participants were tested in experiments 1, 2, and 3, respectively. In total, 72 volunteers participated in the study. They were all right-handed and had normal or corrected-to-normal vision and normal hearing. Participants gave their written informed consent and were compensated for their participation. An adequate level of task performance was confirmed, per participant and stimulus type, by checking that the PSE values did not fall outside the range of tested durations [23]. Based on this criterion, the data from three participants in experiment 2 were excluded due to poor duration discrimination performance for visual stimuli (none was excluded from experiments 1 and 3). Therefore, data from 24 participants (20 females; mean age: 19.4 ± 2.0 years) in experiment 1, 21 participants (18 females; mean age: 19.0 ± 0.7 years) in experiment 2, and 24 participants (20 females; mean age: 19.8 ± 2.1 years) in experiment 3 were further analyzed. The PSE distributions for these participants are presented in Additional file 1: Fig. S6. These sample sizes are comparable to those used in similar previous studies [22, 23]. The study was carried out in accordance with the Declaration of Helsinki and was approved by the Ethics Committee of the School of Psychology of Shaanxi Normal University.

### Apparatus and stimuli

All experiments included visual and auditory stimuli. In experiments 1 and 2, the visual stimulus was a symmetrical Gaussian blob (standard deviation, SD = 0.5°, Michelson contrast = 0.7), and the auditory stimulus was a white noise burst (~55 dB sound pressure level, SPL). In experiment 3, the visual stimuli were either solid white circles (radius ~1.2°) or solid white rings (outer radius ~1.3°, inner radius ~0.6°), and the auditory stimuli were 500 Hz and 2000 Hz pure tones at ~62 dB (SPL). All visual stimuli were presented at the center of a 20″ CRT monitor (85 Hz refresh rate, 1600 × 1200 pixels), and all auditory stimuli were presented via headphones. The auditory stimuli were generated with 10-ms cosine on- and off-ramps (included in the total stimulus duration). For experiment 3, we chose circles and rings, because they differ topologically [62], meaning that one cannot be morphed into the other by a continuous deformation. And, according to the topological approach to perceptual organization, the visual system is highly sensitive to topological differences (in this case the presence or absence of a hole) more than differences in shape (e.g., a square can be continuously deformed into a circle). It has been previously shown that people easily distinguish between a circle and a ring [62, 63].

During the experiments, participants sat on a chair with their head held in place by a chin rest at a distance of 58 cm from the center of the monitor. Participants were instructed to fixate on a red fixation point that was presented at the center of the screen throughout the experiment. Stimulus presentation and data collection were implemented by custom computer programs designed with Matlab and Psychophysics Toolbox extensions [66, 67].

### Procedure

The modified temporal bisection task was used (Fig. 1). Unlike the original temporal bisection task, where participants are asked to judge whether the duration of a test stimulus is closer to a short or long reference stimulus duration, the modified version asks participants to judge whether the test duration is shorter or longer vs. an intermediate reference stimulus [24].

### Experiment 1

All participants completed both visual and auditory tasks, in separate blocks, counterbalanced across participants. Each trial started with a 500-ms blank period (with no stimulus, besides the fixation point). Then, a visual or auditory test stimulus was presented. The duration of the test stimulus was one of five logarithmically spaced intervals of time, between 300 and 900 ms (i.e., 300, 395, 520, 684, 900 ms). Before beginning each block, participants were presented with five or more visual (or auditory) reference stimuli with duration 520 ms (the geometric mean of the test stimuli durations). They were instructed to remember the reference duration and to make subsequent judgments regarding test stimuli relative to the reference duration (the PSE values in Additional file 1: Fig. S6 confirm that participants indeed complied with this instruction). Before beginning the task, participants were allowed to continue to experience the reference stimulus repeatedly, until they were confident that they had remembered the reference duration. Once the block began, participants could no longer experience the reference stimulus.

Participants reported their choice on each trial after the stimulus had ended by pressing one of two keyboard buttons. The button-choice contingency was counterbalanced across participants: half the participants pressed the “F” button with their left index finger and the “J” button with their right index finger to indicate longer and shorter test durations, respectively, while the other half responded with the reverse mapping. Participants were instructed to make each response as quickly and accurately as possible. To ensure an equal number of consecutive stimulus pairs, while retaining a long-term pseudorandom structure, the order of stimulus presentation was determined by a pseudorandom 5^4^ de Bruijn sequence. This creates an optimally short (pseudorandom) sequence of stimuli (625 trials total) in which each contiguous subsequence of 4 stimuli (from the 5 possible stimulus intervals) occurs exactly once [68]. Accordingly, each individual stimulus occurred 125 times, each possible pair occurred 25 times, each possible triplet occurred 5 times, and each quadruplet occurred once (per block).

### Experiment 2

The procedure was similar to experiment 1, with the following differences. In experiment 2, all participants completed one block that comprised both visual and audio trials, interleaved. During the task, either a visual or an auditory stimulus was presented on each trial. Two sensory modalities × five stimulus durations yielded 10 types of stimuli. The order of stimulus presentation was determined by a pseudorandom 10^3^ de Bruijn sequence consisting of 1000 trials, in which each individual stimulus occurred 100 times, each possible pair occurred 10 times, and each possible triplet occurred once [68]. Before beginning the task, participants experienced five or more alternating presentations of visual and auditory stimuli with 520-ms duration. They were asked to remember the visual and auditory reference durations and to make subsequent judgments about the test stimuli relative to the corresponding references. That is, participants needed to compare the visual test with the visual reference and to compare the auditory test with the auditory reference.

### Experiment 3

In experiment 3, each participant needed to complete the two (visual and auditory) tasks in separate blocks. The order of the two blocks was counterbalanced across participants. The procedure of each task (block) was similar to experiment 2 except that different stimuli from the same modality were used. In the visual task, a circle or a ring was presented in each trial; in the auditory task, a high-frequency or a low-frequency tone was presented in each trial. At the beginning of each task, participants experienced five or more alternating presentations of the stimuli (circle and ring in the visual task; high-frequency and low-frequency tones in the auditory task) with 520-ms duration. We asked participants to remember the reference duration of each stimulus and to make subsequent judgments on test stimuli relative to the corresponding references in each task.

### Analysis

For each participant and condition (see condition details in Table 1), trials with RTs larger than 3 median absolute deviations (MADs) from the median were excluded from the analysis [69]. This excluded 6.6% of trials in experiment 1, 6.3% of trials in experiment 2, and 7.2% of trials in experiment 3.

### Psychometric analysis

A psychometric function was defined by the proportion of longer choices as a function of the logged test durations. A cumulative Gaussian distribution was fitted to the data, per participant and condition, using the psignifit toolbox (version 4, see https://github.com/wichmann-lab/psignifit/wiki/ [70]). The PSE was defined by the mean of the fitted cumulative Gaussian distribution function. The PSE corresponds to the duration (log value) that the subject would have equally likely judged as longer or shorter (in some previous studies this is called the “bisection point”). The Weber ratio (WR) was used to quantify temporal sensitivity. This was defined by the perceptual threshold (SD of the fitted cumulative Gaussian) normalized by the PSE. A smaller WR reflects higher temporal sensitivity.

For consistency and comparison to previous studies, we first applied similar methods, which sort the trials conditioned on previous stimuli or previous choices. However, it is important to note that the previous stimulus and previous choice effects are not well separated in this analysis (see explanation in the section “Psychometric results” in the “Results” section). We present these psychometric results for experiment 1 in Additional file 1: Figs. S1 and S2.

For this analysis, the trials were sorted into five groups, according to the five possible previous test durations, and each was fitted with a separate psychometric. Next, we fitted a linear regression of the PSE values vs. the previous stimulus durations (also using log values, like the PSEs) and took the slope to represent the change in PSE by changes in previous stimulus duration. For consistency with other measures (described below), we used the negative slope value (i.e., we flipped the sign), such that positive and negative values represent attractive and repulsive effects, respectively (we refer to this measure, of the change in PSE by previous stimulus duration, by: $\frac{\delta \textrm{PSE}}{\delta \left(\textrm{prev}\_\textrm{stim}\right)}$). Comparably, the trials were sorted into two groups, according to the previous trials’ choices (longer or shorter), which were fitted with separate psychometrics. The difference in PSE between the groups with shorter and longer prior choices (ΔPSE_prev_choice_) was then calculated, to reflect the change in PSE, by prior choices. Both $\frac{\delta \textrm{PSE}}{\delta \left(\textrm{prev}\_\textrm{stim}\right)}$ and ΔPSE_prev_choice_ are unitless (because of log properties [71]). For both of these measures, positive and negative values represent attractive and repulsive effects, respectively. However, as explained above, they cannot be interpreted to reflect independent contributions of previous stimuli and previous choices.

### Probabilistic choice model

To better dissociate the separate effects of previous stimuli and previous choices, we adapted the probabilistic choice model from previous studies [27, 28, 72–74]. Specifically, the model estimates the probability of making a longer response (L) for the current stimulus on trial *t* following the binomial logistic regression:

1 $${p}_t(L)=\frac{1}{1+{e}^{-{z}_t}}$$

where *z*_*t*_ is a linear combination of four predictors that might affect the current perceptual decision (Fig. 2):

2 $${z}_t={\beta}_0+{\beta}_{\textrm{curr}\_\textrm{stimulus}}\times {s}_t+{\beta}_{\textrm{prev}\_\textrm{stimulus}}\times {s}_{t-1}+{\beta}_{\textrm{prev}\_\textrm{choice}}\times {c}_{t-1}$$

Here, *β*_0_ represents the participant’s baseline bias for longer or shorter choices; *s*_*t*_ is the duration of the current stimulus (on trial *t*) and *β*_curr _ stimulus_ is its fitted weight; *s*_*t* − 1_ is the duration of the previous stimulus (on trial *t*-1) and *β*_prev _ stimulus_ is its fitted weight; *c*_*t* − 1_ is the choice from the previous trial and *β*_prev _ choice_ is its fitted weight. In this study, choices were binary (shorter/longer; coded by −1 and +1, respectively), while stimulus durations were logarithmically spaced (between 300 and 900ms). To make these parameters comparable, stimulus durations were logged and then normalized by the root-mean-square (RMS) so that both the previous stimulus duration and choice predictors had RMS = 1.

To assess the effects of the predictors on the current perceptual choice, four models were considered and compared. These models were based on the same logistic regression. They differed only by their input parameters. Specifically, (a) *M*_0_ (no-history model) is the basic model, only including the baseline bias and current stimulus (*β*_prev _ stimulus_ and *β*_prev _ choice_ from Eq. (2) are set to zero); (b) *M*_1_ (stimulus history model) is like *M*_0_, but also takes the previous stimulus into account (*β*_prev _ choice_ from Eq. (2) is set to zero); (c) *M*_2_ (choice history model) is like *M*_0_, but also takes the previous choice into account (*β*_prev _ stimulus_ from Eq. (2) is set to zero); (d) *M*_3_ (stimulus and choice history model) takes into account all the predictors from Eq. (2) (no coefficients are set to zero).

The AIC was used to compare the models. The AIC for a given model is defined by:

3 $$\textrm{AIC}=-2\log \left(\hat{L}\right)+2k$$

where *k* is the number of parameters in the model and $\hat{L}$ is its maximum likelihood. AIC values were calculated for each model and each participant. We then calculated the arithmetic difference between the models’ AIC values (ΔAIC):

4 $$\Delta \textrm{AIC}={\textrm{AIC}}_{M_i}-{\textrm{AIC}}_{M_0}$$

where *M*_*i*_ represents one of the history models (*M*_1_, *M*_2_, or *M*_3_) being compared to *M*_0_ (no-history model).

Because AIC can be biased in favor of complex models, we also compared the models using the BIC, which penalizes model complexity more heavily. The BIC is defined by:

5 $$\textrm{BIC}=-2\log \left(\hat{L}\right)+k\log (n)$$

where *k* and $\hat{L}$ are the same parameters from Eq. 3 and *n* is the number of observations. The arithmetic difference between the models’ BIC values (ΔBIC) was calculated, similar to ΔAIC (Eq. 4).

A lower AIC (BIC) value means a more likely model. ΔAIC (ΔBIC) magnitudes between 2 and 6, between 6 and 10, and > 10 are considered positive, strong, and very strong evidence for the model with the lower AIC (BIC) value [75]. Moreover, to test the necessity of the extra parameters of the history models, we used the likelihood ratio test to compare the history models (*M*_1 − 3_) to the no-history model (*M*_0_). Specifically, we used history models as the unrestricted models and the no-history model as the restricted (nested) model. For each participant and condition, we compared the unrestricted model against the restricted model and calculated the proportion of participants for which the history model (or no-history model) provided better fits.

### Statistics

Statistical analyses were performed using JASP (version 0.16.1.0) and Matlab. Analyses of the PSE shifts, conditioned on the previous trials, yielded two measures ($\frac{\delta \textrm{PSE}}{\delta \left(\textrm{prev}\_\textrm{stim}\right)}$ and ΔPSE_prev_choice_) per sensory modality (visual and auditory) in experiment 1. Firstly, we tested whether each measure was significantly different from zero (using one-sample two-tailed *t*-tests; two *t*-tests per modality). Secondly, we examined whether these measures differed by sensory modality (using two paired-samples two-tailed *t*-tests). Because each dataset was used for four comparisons, we multiplied the *p*-values by four (Bonferroni correction).

For the model statistics, we focused on two parameters: *β*_prev _ stimulus_ and *β*_prev _ choice_ which were fitted simultaneously using *M*_3_ (stimulus and choice history model). These parameters reflect the separate effects of previous stimuli and previous choices, respectively. The other two model parameters (*β*_0_ and *β*_curr _ stimulus_) are unrelated to trial history and were tested for sanity checks only (namely, to confirm a strong influence of the current stimulus, and to check for any systematic baseline biases; see Additional file 1: Fig. S7). In experiment 1, the two measures (*β*_prev _ stimulus_ and *β*_prev _ choice_) were compared, per sensory modality (visual and auditory). Firstly, we tested whether each measure was significantly different from zero (using one-sample two-tailed *t*-tests; two *t*-tests per modality). Secondly, we examined whether these measures differed by sensory modality (using two paired-samples two-tailed *t*-tests). Because each dataset was used for four comparisons, we multiplied the *p*-values by four (Bonferroni correction).

In experiments 2 and 3, the data for each condition (see Table 1 parts B and C) were fitted using *M*_3_ (stimulus and choice history model), similar to experiment 1. We first tested whether the history-related coefficients (*β*_prev _ stimulus_ and *β*_prev _ choice_) were different from zero (per condition) using one-sample two-tailed *t*-tests. Then, for experiment 2, we conducted a two-way repeated-measures ANOVA for each coefficient (*β*_prev _ stimulus_ and *β*_prev _ choice_), to test the effects of modality (visual or auditory) and context (uni-modal or cross-modal). For experiment 3, similar ANOVAs were used to test the effects of stimulus (circle or ring for visual; high-pitch or low-pitch for auditory) and context (consistent or inconsistent stimulus). The *p*-values resulting from these comparisons were multiplied by four (Bonferroni correction) considering that each dataset was used in four statistical comparisons in each experiment.

## Supplementary Information

**Additional file 1: Supplementary Results.** Psychometric results (Experiment 1) – further details. **Figure S1**. Psychometric analyses for Experiment 1. **Figure S2**. Weber ratio (WR) values from Experiment 1. **Figure S3**. BIC model comparisons. **Figure S4**. Likelihood ratio tests. **Figure S5**. Psychometric analyses for Experiments 2 and 3. **Figure S6**. Distribution of PSE values across participants per stimulus type. **Figure S7**. Non-history-related model parameters.

## Data Availability

All data generated or analyzed during this study are included in this published article, its supplementary information files, and publicly available repositories (https://osf.io/cnptb/) [76].

## Abbreviations

SC: Sensory carryover
DC: Decisional carryover
PSE: Point of subjective equality
AIC: Akaike information criterion
BIC: Bayesian information criterion
ANOVA: analysis of variance
IRM: Internal reference model
SMG: Supramarginal gyrus
SMA: Supplementary motor area
PPC: Posterior parietal cortex
PFC: Prefrontal cortex
CGp: Posterior cingulate cortex
FEF: Frontal eye field
dlPFC: Dorsolateral prefrontal cortex
SD: Standard deviation
SPL: Sound pressure level
MAD: Median absolute deviation
WR: Weber ratio
RMS: Root-mean-square
