# Supplementary material for: Modality-specific sensory and decisional carryover effects in duration perception
Source: BMC Biol. 2023 Mar 8;21:48. doi: 10.1186/s12915-023-01547-9 (PMC9993637; doi:10.1186/s12915-023-01547-9)
Supplement: Supplementary file 1 — Additional file 1: Supplementary Results. Psychometric results (Experiment 1) – further details. Figure S1. Psychometric analyses for Experiment 1. Figure S2. Weber ratio (WR) values from Experiment 1. Figure S3. BIC model comparisons. Figure S4. Likelihood ratio tests. Figure S5. Psychometric analyses for Experiments 2 and 3. Figure S6. Distribution of PSE values across participants per stimulus type. Figure S7. Non-history-related model parameters. [file 12915_2023_1547_MOESM1_ESM.pdf]

Supplementary Material

Modality-specific sensory and decisional carryover effects in duration perception

Baolin Li[1*], Biyao Wang[1], Adam Zaidel[2]

[1]School of Psychology, Shaanxi Normal University, 199 Chang'an South Road, Yanta District, Xi'an 710062, China

[2]Gonda Multidisciplinary Brain Research Center, Bar-Ilan University, Ramat Gan, 5290002, Israel

*For correspondence: lblpsy@snnu.edu.cn

Supplementary Results

Psychometric results (Experiment 1) – further details

Psychometric plots, with the data sorted by previous stimuli or by previous choices are presented in Fig. S1. These are shown for an example participant (panels A and B) as well as averaged data across all participants (panels C and D). At the group level (with psychometric curves fit per participant), the change in PSE by previous stimuli ($\frac{\delta \text{PSE}}{\delta(\text{prev\_stim})}$) was not significantly different from zero, neither for visual nor auditory stimuli (Fig. S1, E; statistics presented in the main manuscript). However, the changes in PSE by prior choices ($\Delta \text{PSE}_{\text{prev\_choice}}$) were significantly larger than zero for both visual and auditory stimuli (Fig. S1, F; statistics presented in the main manuscript). When comparing modalities, no significant difference in $\frac{\delta \text{PSE}}{\delta(\text{prev\_stim})}$ was seen between vision and audition ($t(23) = 0.5$, $p = 1.00$, Cohen's $d = 0.11$). However, $\Delta \text{PSE}_{\text{prev\_choice}}$ was significantly larger in vision vs. audition ($t(23) = 3.3$, $p = 0.013$, Cohen's $d = 0.67$). These results should be treated with caution due to the limitations of this method.

In exploratory analyses, we investigated the effects of previous stimuli and previous choices on the Weber ratio (WR, Fig. S2). We did not observe any significant effects of previous stimuli or previous choices on temporal sensitivity, but replicated a well-known finding of superior auditory (vs. visual) performance in temporal processing [41-43].

Supplementary Figures

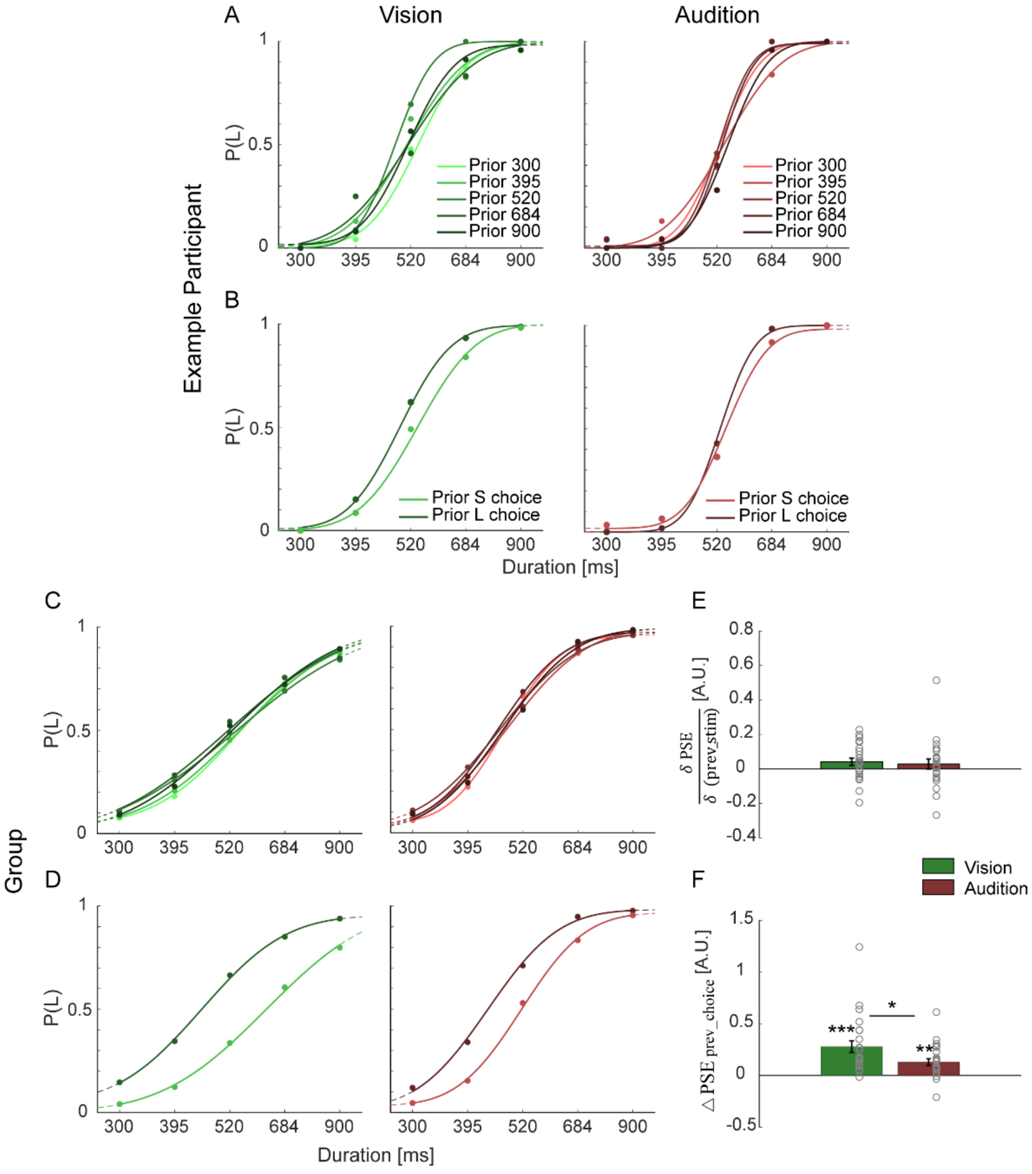

Figure S1. Psychometric analyses for Experiment 1. (A) Example psychometric data from a naive, representative, observer. Vision and audition data are presented in shades of green and maroon, respectively. The data-points (and psychometric fits) reflect the proportion of 'longer' choices, as a function of stimulus duration on the current trial. Five separate psychometric plots are presented, for the data sorted according to the five possible previous stimulus durations (prior 300, 395, 520, 684 or 900 ms). (B) The same data from (A), but sorted according to the previous trials' choices (prior S or L). (C, D) Average psychometric data across all observers sorted by (C) previous stimulus durations and (D) previous choices (like A and B, respectively). The x-axes for the psychometric plots (A, B, C and D) are in log scale. (E) Group results for the measured change in PSE by change in previous stimulus duration, $\frac{\delta \text{PSE}}{\delta(\text{prev\_stim})}$. (F) Group results for the change in PSE by prior choice, $\Delta \text{PSE}_{\text{prev\_choice}}$. In E and F, bars represent group means, error bars indicate standard errors of the mean and gray circles represent individual participants' values. *** $p < 0.001$, ** $p < 0.01$, * $p < 0.05$, Bonferroni corrected by multiplying the raw p-values by four.

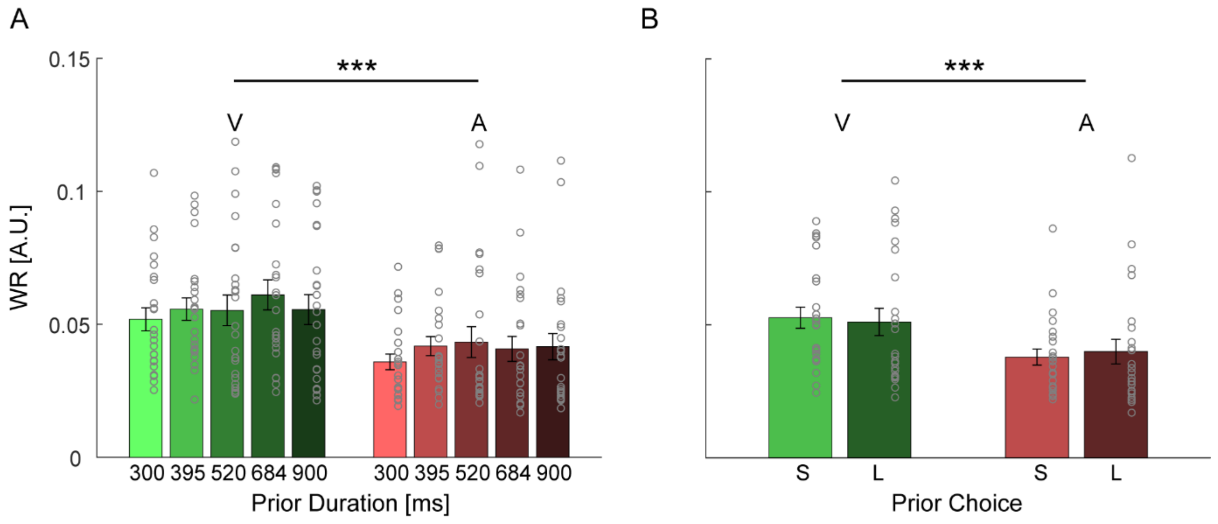

Figure S2. Weber ratio (WR) values from Experiment 1. (A) WR separated by prior duration (300, 395, 520, 684, and 900 ms) and sensory modality (V: vision; A: audition). (B) WR separated by prior choice (S: 'shorter' choice; L: 'longer' choice) and sensory modality (V: vision; A: audition). Bars represent group means, error bars indicate standard errors of the mean and gray circles represent individual participants' values. *** $p < 0.001$ (uncorrected).

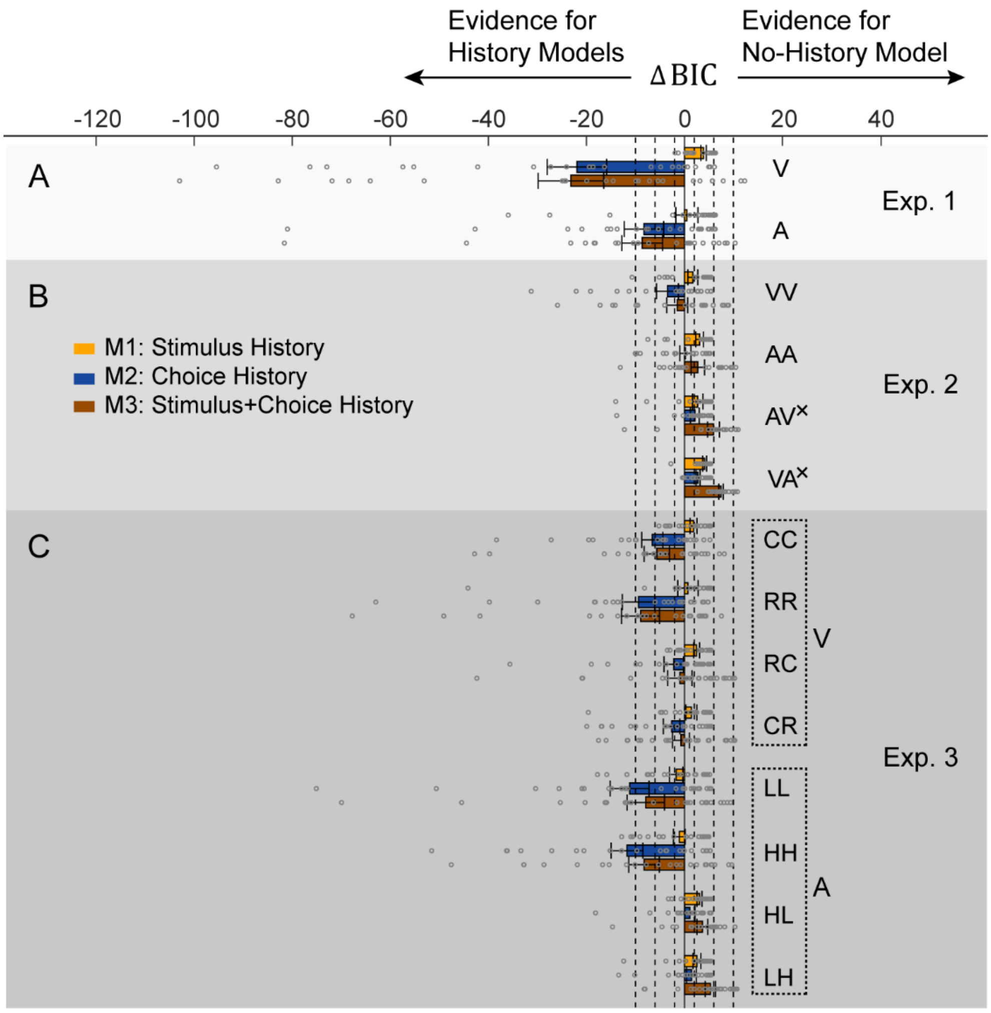

Figure S3. BIC model comparisons. All conventions are the same as in Figure 3 except that the Bayesian information criterion (BIC) was used instead of the Akaike information criterion (AIC).

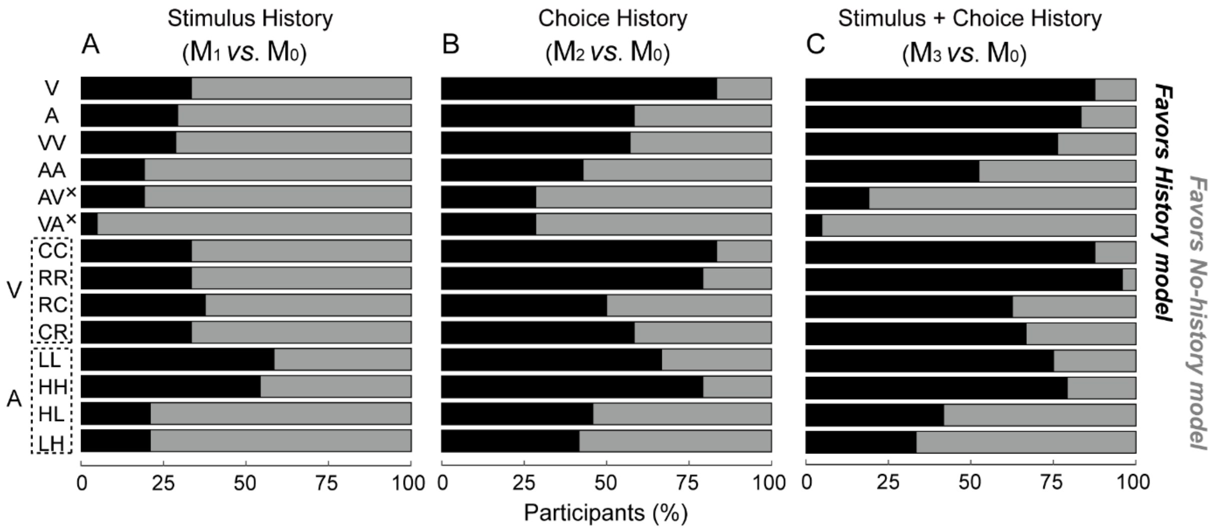

Figure S4. Likelihood ratio tests. Comparisons of (A) M_1 'stimulus-history' model, (B) M_2 'choice-history' model and (C) M_3 'stimulus and choice history' model to M_0 'no-history' model. Black (and complementary gray) bars reflect the proportion of participants for which the history model (and no-history model) provided better fits according to a likelihood ratio test, per condition (rows). Conditions are marked on the left of the plot by one- or two-letter codes (explained in Table 1; $^{\times}$marks cross-modal conditions).

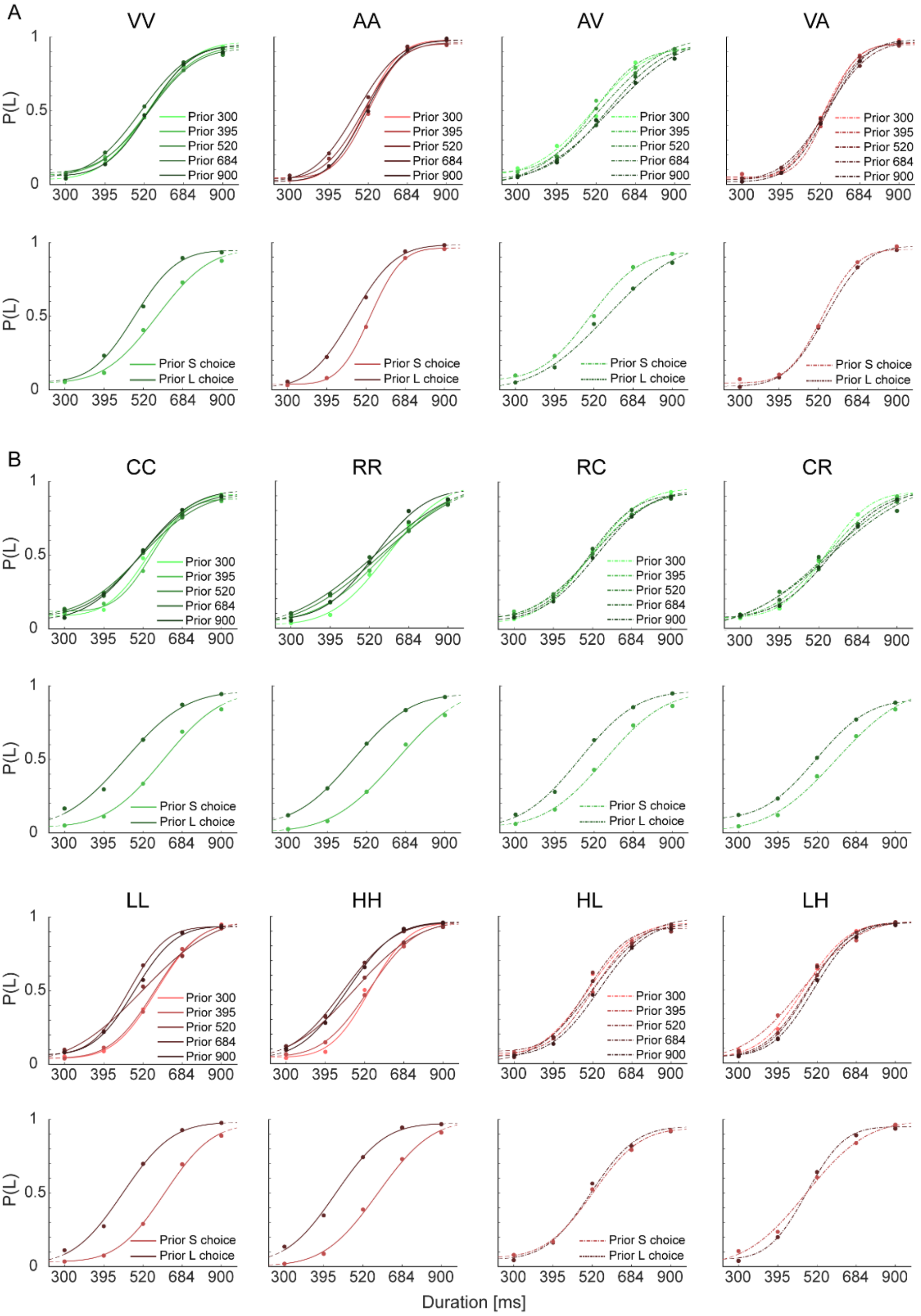

Figure S5. Psychometric analyses for Experiments 2 and 3. Average psychometric plots (data sorted by prior stimuli or prior choices, and averaged across participants) are presented for (A) Experiment 2, and (B) Experiment 3. Conditions are marked by one- or two-letter codes (explained in Table 1B, C). The x-axes are in log scale. We note that this analysis can misrepresent results. For example, in the consistent-stimulus context (i.e., conditions CC, RR, LL and HH), for which logistic model fits found attractive (positive) DC and repulsive (negative) SC, this psychometric analysis (incorrectly) shows attractive (positive) SC. This is due to the unbalanced distribution of prior choices when sorting the data by prior stimulus duration (see the manuscript for further details).

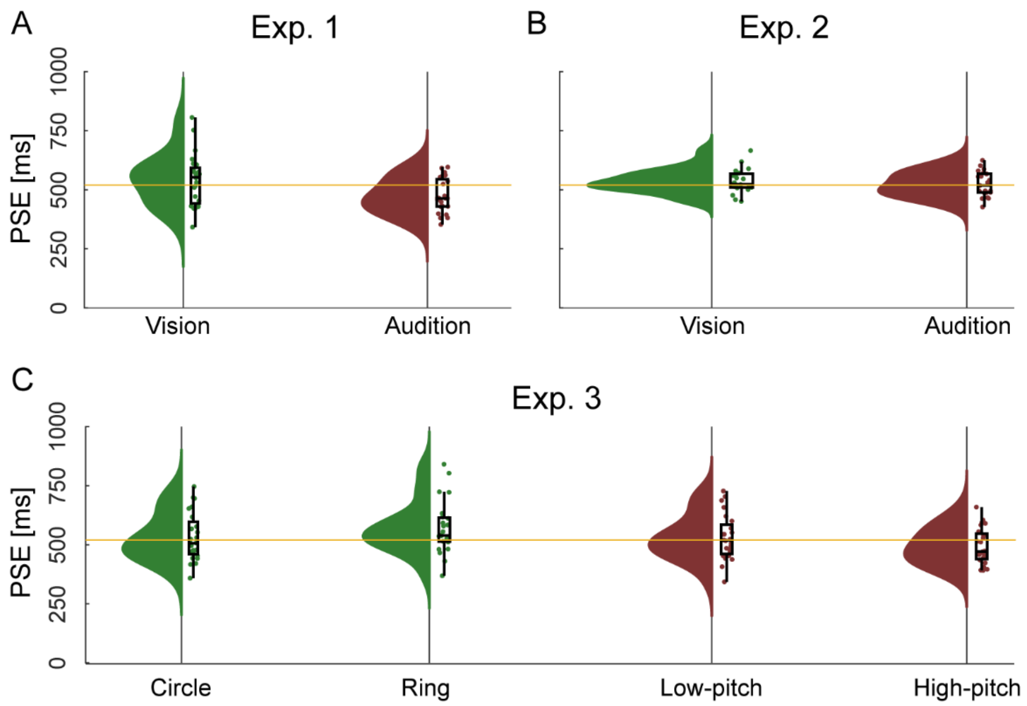

Figure S6. Distribution of PSE values across participants per stimulus type. (A) Experiment 1, (B) Experiment 2, and (C) Experiment 3. The PSEs were calculated by pooling all the same stimulus trials for a participant (ignoring history effects). Distributions are presented in green and maroon for visual and auditory stimuli, respectively. Each dot represents the PSE for one participant, superimposed on box plots (which present median and interquartile ranges). The orange horizontal line represents the reference duration (520 ms).

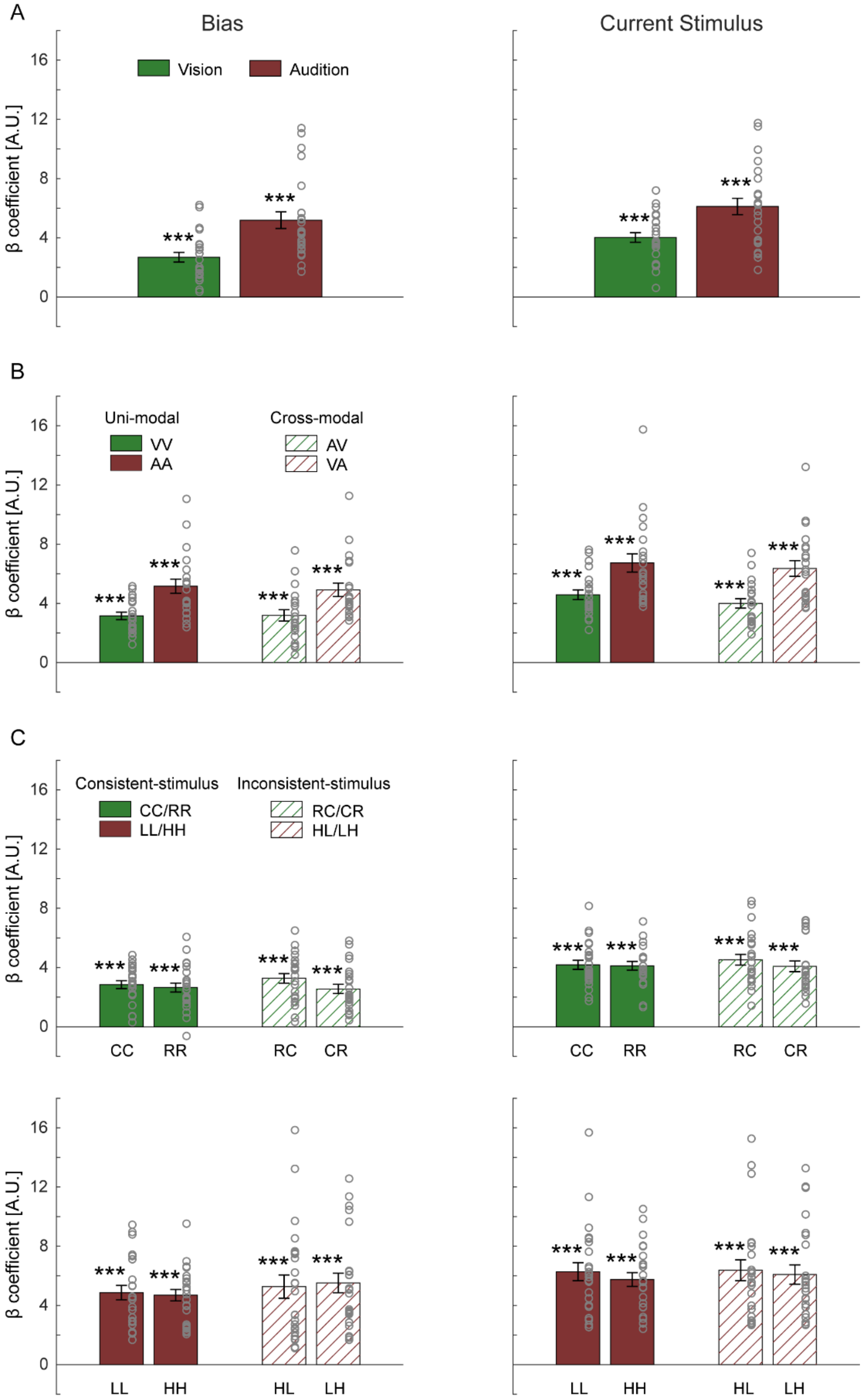

Figure S7. Non-history-related model parameters. Beta coefficients for the bias (β_0, left column) and current stimulus ($\beta_{curr\_stimulus}$, right column) from the M_3 ('stimulus and choice history') model fits in the different conditions of (A) Experiment 1, (B) Experiment 2 and (C) Experiment 3. See condition details in Table 1. Bars represent group means, error bars indicate standard errors of the mean and gray circles represent individual participants' values. *** p < 0.001, Bonferroni corrected by multiplying the raw p-values by four.
